# A High Throughput Screening HPLC-FLD Method for Paralytic Shellfish Toxins (PSTs) Enabling Effective Official Control

**DOI:** 10.3390/molecules27154702

**Published:** 2022-07-22

**Authors:** Melania Siracusa, Simone Bacchiocchi, Alessandra Dubbini, Debora Campacci, Tamara Tavoloni, Arianna Stramenga, Martina Ciriaci, Sonia Dall’Ara, Arianna Piersanti

**Affiliations:** 1 Istituto Zooprofilattico Sperimentale Umbria e Marche “Togo Rosati”, Via Cupa di Posatora 3, 60131 Ancona, AN, Italy; m.siracusa@izsum.it (M.S.); s.bacchiocchi@izsum.it (S.B.); d.campacci@izsum.it (D.C.); t.tavoloni@izsum.it (T.T.); a.stramenga@izsum.it (A.S.); m.ciriaci@izsum.it (M.C.); 2 Fondazione Centro Ricerche Marine, Viale A. Vespucci 2, 47042 Cesenatico, FC, Italy; sonia.dallara@centroricerchemarine.it

**Keywords:** paralytic shellfish toxins, HPLC-FLD, official control, screening method, marine biotoxins, gastropods

## Abstract

Paralytic Shellfish Toxins (PSTs) are marine biotoxins, primarily produced by dinoflagellates of the genera *Gymnodinium* spp., *Alexandrium* spp. They can accumulate in shellfish and, through the food chain, be assimilated by humans, giving rise to Paralytic Shellfish Poisoning. The maximum permitted level for PSTs in bivalves is 800 μg STX·2HCl eqv/kg (Reg. EC N° 853/2004). Until recently, the reference analytical method was the Mouse Bioassay, but Reg. EU N° 1709/2021 entered into force on 13 October 2021 and identified in the Standard EN14526:2017 or in any other internationally recognized validated method not entailing the use of live animals as official methods. Then the official control laboratories had urgently to fulfill the new requests, face out the Mouse Bioassay and implement instrumental analytical methods. The “EURLMB SOP for the analysis of PSTs by pre-column HPLC-FLD according to OMA AOAC 2005.06” also introduced a simplified semiquantitative approach to discriminate samples above and below the regulatory limit. The aim of the present paper is to present a new presence/absence test with a cut-off at 600 μg STX·2HCl eqv/kg enabling the fast discrimination of samples with very low PSTs levels from those to be submitted to the full quantitative confirmatory EN14526:2017 method. The method was implemented, avoiding the use of a large number of certified reference standards and long quantification procedures, resulting in an efficient, economical screening instrument available for official control laboratories. The protocol was fully validated, obtaining good performances in terms of repeatability (<11%) and recovery (53–106%) and accredited according to ISO/IEC 17025. The method was applied to mollusks collected from March 2021 to February 2022 along the Marche region in the frame of marine toxins official control.

## 1. Introduction

**Figure 1:**
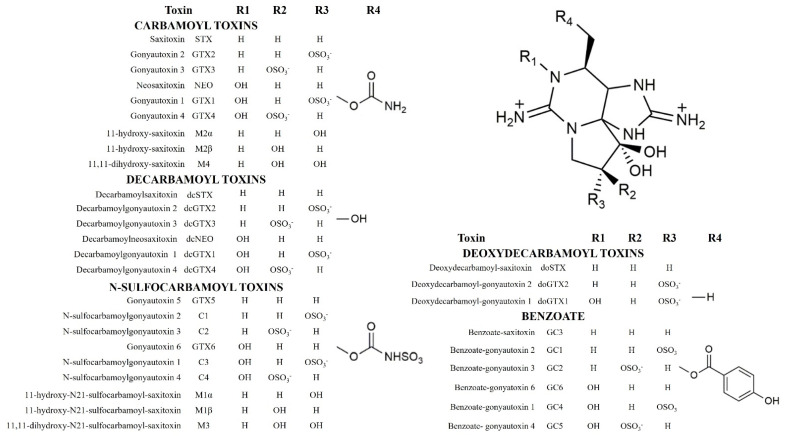
Chemical structures of the PST toxins identified and reported in literature. PSTs are produced by marine microalgae, specifically dinoflagellates of the genera.

Paralytic Shellfish Toxins (PSTs) are biotoxins naturally occurring in marine and freshwater environments. They are a group of water-soluble neurotoxic alkaloids, which include different tetrahydropurine analogs resistant to heat and acid conditions [1]. The first PST discovered was Saxitoxin (STX), a heterocyclic guanidine derivative so toxic for humans and animals that it is listed among the possible chemical weapons [2]. More than 50 PSTs analogs have been described to date; they differ in side group moieties and are divided into five classes: (a) carbamoyl-, (b) decarbamoyl-, (c) N-sulfocarbamoyl-, (d) deoxydecarbamoyl-, and (e) benzoate toxins; the latter does not yet have completely clear toxic properties [1,3,4]. The chemical structures of the known PSTs toxins are shown in Figure 1. For fifteen analogs, a Toxicity Equivalency Factors (TEFs) list was compiled by European Food Safety Authority EFSA [2].

*Gymnodinium* spp., *Alexandrium* spp. And *Pyrodinium* spp. The PSTs toxin profile characterizing dinoflagellates differs per algae species and/or per region of occurrence. PSTs accumulate in bivalve mollusks upon microalgae ingestion, and, subsequently, humans may be intoxicated through seafood consumption. Globally, in the last decades, intoxication cases have been reported with a rising trend [1]. Paralytic Shellfish Poisoning (PSP) symptoms include nausea, neuromuscular paralysis, respiratory and cardiovascular distress, and eventual death. According to available studies, the binding of STX to voltage-gated sodium channels α-subunit of muscle and nerve cells blocks ion conductance [2], giving rise to paralysis.

PSTs have been detected in bivalves from all over the world [5,6,7,8]. In Italy, toxic events have been described since 2000s in mussel farms from Sicily and Sardinia, mainly caused by *Alexandrium* species (Dinophyceae) [9]. Very high PSTs levels, never found before in Italy, were measured in mussels collected in 2015–2017 from Syracuse Bay [10]. Conversely, the PSTs levels measured along the Adriatic coast are not a risk for human safety with sporadic detections in Emilia Romagna [11] and in the North-east of the peninsula [12], although other marine biotoxins such as Yessotoxins, Okadaic Acid, and Cyclic Imines were recurrently measured in mussels [13,14].

Regulation (EC) No 853/2004 [15] set the maximum limit (ML) of 800 µg STX·2HCl eqv/kg; then, the European Union requested member states’ continuous PSTs monitoring in bivalve mollusks [16]. Until recently, Mouse Bioassay (MBA) (according to OMA AOAC 959.08) was the official method for PSTs analysis in bivalves [17] and, as a result of its cost-effectiveness and rapidity, it remained the reference confirmatory method until 2018 [18] although Commission Directive N° 63/2010 [19] encouraged the substitution of the biological assay with instrumental analysis, avoiding animals sacrifice.

Meanwhile, chromatographic methods based on pre-column (OMA AOAC 2005.06, also called Lawrence Procedure) or post-column oxidation (AOAC 2011.02) with fluorescence detection were developed and validated through collaborative study [20,21]. Both the methods are long, laborious, and expensive. Recently, methods with Hydrophilic Interaction Liquid Chromatography coupled to mass spectrometry (HILIC-MS) were implemented [22,23,24]. Mass spectrometry will surely be the election analytical technique in the near future as a result of the comprehensive structural information that enables the collection, but now it still does not reaches the necessary sensitivity and reproducibility.

In June 2020, the “EURLMB SOP for the analysis of Paralytic Shellfish Toxins by precolumn HPLC-FLD according to OMA AOAC 2005.06” (from now on called EURLMB SOP) was published by the European Reference Laboratory for Marine Biotoxins [25] describing two different screening approaches in the application of OMA AOAC 2005.06: a presence/absence test and a semiquantitative analysis with 600 µg STX·2HCl eqv/kg cut-off. In 2019, Commission Regulation (UE) N° 627/2019 [26] established that the OMA AOAC 2005.06 was the official reference method for PSTs analysis. Subsequently, in 2021, Commission Regulation (EU) 2021/1709 [27] recognized in the EN 14526:2017 [28] the analytical official reference standard and faced out the MBA since 13 October 2021.

Official Control Laboratories had, therefore, urgently to align to the new legislative prescription switching from MBA to HPLC-FLD since, in the European framework, in the field of official marine biotoxins shellfish control, only official methods should be chosen. Over the years, the Marche coast mussels breeding sites increased, raising the number of samples significantly to be controlled. Since PSTs are not frequently detected in the Adriatic Sea, it was decided to implement a screening instrumental analytical method in order to quickly distinguish negative samples from suspect ones. The same double-stage screening/confirmatory approach was already successfully implemented in 2008 in other analytical fields, validating high throughput, cheaper screening procedures [29].

The present paper describes a new pre-column oxidation presence/absence screening chromatographic method based on the EURLMB SOP from which also the 600 µg STX·2HCl eqv/kg cut-off was borrowed and used as screening target concentration (STC) [30]. This approach significantly reduces the number of samples to be confirmed by the EN 14526:2017, specifically in periods and geographic areas in which PST levels are negligible, avoiding the use of a large number of standards and long quantification procedures resulting furthermore in a sensitive and selective tool. Additionally, the method was also extended to analyze gastropods not included in the aim of EURLMB SOP.

The method was fully validated and accredited according to UNI CEI EN ISO/IEC 17025:2018 [31], and its performances were compared to the confirmatory method (EN14526:2017) and also evaluated by participation in national and international proficiency tests.

Finally, it was applied to one-year monitoring of mollusks collected along the Marche coast (north-central Adriatic Sea, Italy) in the frame of official control.

## 2. Results and Discussion

### 2.1. PSTs Analysis

PSTs are marine biotoxins to be controlled in mussels and bivalves in the frame of monitoring programs to fulfill Regulation (EU) 2017/625 [16] and Regulation (EC) 853/2004 [15] requirements and to safeguard consumer health. In the last ten years, thousands of mussel samples collected in the Adriatic Sea were analyzed for PSTs by MBA in official laboratories, and none of them were identified as positive. Therefore, it can be concluded that, at present, the vast majority of the monitored samples are negative or characterized by PST levels significantly below the regulatory limit. It is, therefore, in most cases, unnecessary to employ the full quantitative, long and laborious EN 14526:2017 [28] procedure to screen out negative samples.

The full EN 14526:2017 method includes an extraction, a double cleanup step using C18 SPE, ion-exchange fractionation, and multiple precolumn derivatizations using both periodate and peroxide. Moreover, for quantification purposes, the number of standards required for a full calibration is very high. The sample processing is extremely tangled, long, and time-consuming, the chromatogram interpretation difficult, and the quantification needs several working hours to report results [32]. A simplified analytical approach enabling fast, reliable, high-throughput results issuing to screen out highly contaminated samples from the not contaminated ones is sure to be encouraged.

The EURLMB SOP details how to perform the presence/absence rapid screening: only C18-SPE cleanup and periodate oxidation. If no peaks are identified in the chromatogram, the sample is negative; otherwise, it should be submitted to the confirmatory analysis. Therefore, any sample showing chromatographic peaks of toxin oxidation products with an S/N ≥ 3.0 should be submitted to full quantitation.

When implementing the EURLMB SOP presence/absence method, it turned out that the obtained chromatograms very rarely were characterized by no peaks, also for negative samples. This implies that a lot of samples should have been submitted for confirmatory analysis, losing the advantage of a screening procedure. From the 1st of March to the 30th of September 2021, 352 samples were analyzed, and 282 had at least one peak in the chromatogram needing further investigation (80% of the samples) while, in the application of the here presented presence/absence test with 600 µg STX·2HCl eqv/kg cut-off, none of the 352 samples were identified as suspect to be confirmed.

Application of screening procedures may be found in the literature since 2013, when Harwood et al. [7] and Turner et al. [8] proposed semiquantitative methods with a screening cut-off of 400 µg STX·2HCl eqv/kg. The Harwood and Turner semiquantitative tests require, nevertheless, quantification and ranking of samples in three different categorized groups to which different actions should be applied. Those methods have a less straightforward interpretation with respect to the presented presence/absence test with the cut-off.

The here presented screening method wants to be as easy as possible; therefore, it is run like a presence/absence test but with a threshold cut-off of 600 µg STX·2HCl eqv/kg. In its implementation, no specific quantification is required, and all the tests are interpreted by comparing the chromatographic signals obtained in the samples with the chromatographic signals obtained for the quality control sample spiked at the cut-off (QC+). Therefore, compliant is all the samples in which the sum of PSTs < 600 STX·2HCl eqv/kg, while samples in which PSTs ≥ 600 STX·2HCl eqv/kg should be submitted to confirmation analysis.

Fourteen are the target molecules included in the PST group, and, in the here described approach, seven groups of co-eluting toxins were identified, and seven molecules were taken as representative toxins.

### 2.2. Validation

Method validation was accomplished following Commission Regulation (EU) N^o^ 519/2014 [30], in which the concept of screening target concentration (STC) is introduced. STC is the concentration of interest for the detection of the toxins in a sample. In the implemented screening method, the 600 µg STX·2HCl eqv/kg of the semiquantitative EURLMB SOP was chosen as the threshold and is the STC for the here described presence/absence test; therefore, the aim of the validation study was not the definition of the STC (Commission Regulation (EU) N° 519/2014), but rather the assessment of the implemented method performances and the verification of their compliance to screening method requirements. The presented screening method is used for the selection of samples with a PST level that exceed the STC from the negatives with a given certainty. Generally, for screening methods, a certainty of 95% is considered fit-for-purpose, and a “negative sample” is a sample in which the PSTs content is <STC and the chance that the sample can be incorrectly reported as negative is 5%.

Table 1 summarizes the results obtained during the validation study conducted on mussels and gastropods; data regarding acquired signals and recoveries are ported in Tables S1 and S2. The RSDr for all the seven toxins are all below 11%, demonstrating good repeatability of the method. Reproducibility is significantly worse, reaching 50% in the case of dcSTX. The reason for the scarce reproducibility may be found in the periodate derivatization reaction, which seems to be difficult to repeat from batch to batch, as shown from the signal registered during the validation study for the single toxins (Table S1), representing the main disadvantage of the implemented approach.

Several experiments were tempted aiming to standardize the periodate oxidation reaction (exact oxidant timing, daily preparation of the oxidant solution, exact oxidant pH adjustment at 8.20 ± 0.05), but none of them yielded the desired reproducibility. The RSD_R_ obtained were never better than the figures reported above. In order to tackle this issue, intra-batch management of the analysis and of the control samples was implemented. Therefore, no absolute reference signals were taken for the positive control sample (QC+), but the QC+ was prepared and processed each time within the sample batch; therefore, the signal comparisons between QC+ and samples in the analysis were always within the batch, never from one batch to the other.

Therefore, only the RSDr were used to assess the fit-for-purpose of the PSTs screening method. We should consider the mean RSDr obtained during the validation study to derive all the calculations: 7.6% (Table 1). The degrees of freedom of the validation experiments were calculated considering that in each batch, five independent replicated analyses were performed on five different days (N = 25 independent experiments in reproducibility conditions). The cover factor (which is a multiplicative factor for the RSDr) was calculated from the t-student, taking into account a certainty of 99% (*p* = 0.01) and the 20 degrees of freedom, obtaining t = 2.528. Therefore, the experimental STC was derived by applying the following equation [30]:

(1) $$\mathrm{STC}\;\left(µg\;\mathrm{STX}\;\cdot \;2\mathrm{HCl}\;\mathrm{eqv}/\mathrm{kg}\right)=\mathrm{ML}-\left(\frac{\mathrm{ML}\times t\times {\mathrm{RSD}}_{r}\%}{100}\right)=800-\left(\frac{800\times 2.528\times 7.6}{100}\right)=\;646\;µg\;\mathrm{STX}\;\cdot \;2\mathrm{HCl}\;\mathrm{eqv}/\mathrm{kg}$$

The STC, calculated from the established ML (EURLMB, 2020), taking into account the precision of the method, gives, as a result, 646 µg STX·2HCl eqv/kg, which, in fact, is slightly higher than the STC set by the semiquantitative EURLMB SOP. Therefore, the adoption of a cut-off = 600 µg STX·2HCl eqv/kg assures a more precautionary approach. The method was also tested on gastropods in repeatability conditions (5 samples in one day), and the RSDr were compared to those obtained for mussels by the F-test (data not reported), obtaining positive results: the variability of the method in the two matrices are comparable. Therefore the same STC of 600 µg STX·2HCl eqv/kg may also be used for gastropods. In summary, performing the test as described, given the performances of the here presented screening method, a “negative sample” is a sample in which the PSTs content is <600 µg STX·2HCl eqv/kg and the chance that the sample is incorrectly reported as negative is 1%.

### 2.3. Quality Control

Table 2 reports the results obtained applying the PSTs screening method described by participating in four different inter-calibration exercises.

All the obtained results were compliant with the assigned values except for sample X of the NRLMB March 2021, in which, although being the result significantly lower than 600 μg STX·2HCl eqv/kg, the sample was sent to confirmatory analysis. This happens because of the extremely precautionary approach, which dictates sending the sample to confirmatory analysis if even one only chromatographic peak has a signal 50% larger than the signal obtained for the same toxin in the QC+ sample. This is because it is generally accepted by the scientific community that single-point quantification is allowed only when the sample signal lies between ±50% of the standard reference signal. Therefore, when the sample shows a signal out of the above range, no single-point quantification is allowed. The developed approach is built for application in case of negligible PSTs levels. Anyway, it is fit for this purpose because, in screening methods, false suspect samples are expected.

In QUASIMEME and NRLMB March 2021, a quantitative estimation of the concentrations was given in order to compare the obtained results with the results assigned by the organizers. Furthermore, in the estimation, the screening method behaves quite well, usually overestimating the concentrations, yielding positive z-scores, which is good for a screening method.

Anyway, all the other results were correctly assigned, demonstrating a fit for the purpose of the implemented method.

### 2.4. Field Samples

The PST screening method was accredited at the end of February 2021, and since then, it has been used by the laboratory in all the mussels monitoring programs. Even if all the samples were screened with the here presented presence/absence method with the cut-off set at 600 µg STX·2HCl eqv/kg, we verified that for each of the seven representative toxins, at least a LOQ of 40 µg/kg could be reached. All the 569 samples analyzed were negative: the PSTs were <600 µg STX·2HCl eqv/kg. Anyway, in 14 negative samples, peaks corresponding to GTX 2,3 and GTX 1,4 were identified from the retention time. To better understand whether the peaks were correctly assigned or not, the extracts were sent to the NRL-MB, which re-analyzed the samples with the EN 14526:2017, measuring only in seven of them traces of GTX 2,3 below the method LOQ (=23 µg STX·2HCl eqv/kg). The measured concentrations ranged between 7 and 11 µg STX·2HCl eqv/kg upon the tentative estimation of the levels, even if below the LOQ.

Moreover, six completely negative mussel samples were sent to NRL-MB for confirmation of the presence/absence screening results: none of the toxins was identified in the samples also analyzed by EN 14526:2017. Interestingly all the seven mussels in which traces of GTX 2,3 and GTX 1,4 were identified were sampled in September 2021, suggesting a possible accumulation period in the late summer.

## 3. Materials and Methods

### 3.1. Chemicals and Materials

Acetonitrile (ACN, HPLC-MS grade) and methanol (MeOH, HPLC grade) were purchased from CARLO ERBA Reagents S.r.l. (Milano, Italy). Acetic acid (CH3COOH, pur. ≥ 99.8%), ammonium formate (HPLC grade), sodium hydroxide (NaOH, for analysis grade), and periodic acid (for analysis grade) were obtained from Sigma-Aldrich (Steinheim, Germany). Disodium hydrogenphosphate anhydrous (analysis grade) was obtained from CHEM-LAB NV (Zedelgem, Belgium). Ultrapure water (0.054 µS at 25 °C) was obtained from a MilliQ water purification system (Millipore Ltd., Bedford, MA, USA). STX, GTX1,4, C1,2, dcSTX, dcNEO, GTX2,3, GTX5, NEO, GTX6, and dcGTX2,3 certified solutions were purchased from CIFGA (Lugo, Spain). Stock PSTs standard solutions were prepared in 0.03 M acetic acid. C18-SPE cartridges (Supelclean 3 mL, 500 mg) were purchased from SUPELCO—Sigma-Aldrich (Steinheim, Germany). Hydrogen peroxide (analysis grade) and ammonium acetate (analysis grade) were obtained from VWR International S.r.l. (Milano, Italy). Potassium hydroxide (analysis grade) was purchased from ACROS ORGANICS- Thermo Fisher Scientific (Geel, Belgium). COOH-SPE cartridges (3 mL, 500 mg) were purchased from J.T.BAKER-Thermo Fisher Scientific (Geel, Belgium).

### 3.2. PSTs Extraction and HPLC-FLD Analysis

#### 3.2.1. Presence/Absence Screening with Cut-Off Approach

The test enables the screening of the 14 target PSTs included in the EURLMB SOP. The applied method includes the simplifications of the screening procedure proposed by EURLMB SOP [25], therefore a single purification on C18 SPE (500 mg/3 mL), and only the periodate oxidation reaction.

Briefly, 5.0 ± 0.1 g of the mussel homogenate are weighted in a 50 mL PP tube, 3 mL of acetic acid 1% (*v*/*v*) solution are added to the tube, and the sample is thoroughly vortex shaken and boiled in a water bath for 5 min. The tubes are let cool down to room temperature, vortex shaken, and centrifuged at 4000× *g* for 10 min. The supernatant is withdrawn, and the solid residue is re-extracted a second time in the same conditions without the boiling step. The combined extracts are then brought to 10 mL with water. One of the 10 mL is purified on the C18 SPE. The SPE column is conditioned with 6 mL MeOH and 6 mL H_2_O; the sample is loaded, and, finally, the column eluted with 2 mL of H_2_O. The eluate pH is brought to 6.5 ± 0.2 and diluted to 4 mL with H_2_O. The oxidation is accomplished dispensing 100 µL of the sample/standard in a 2 mL autosampler vial, 100 µL of matrix modifier, vortex shaking, adding 500 µL of periodate oxidant solution (periodic acid 0.03 M/ammonium formiate 0.3 M/disodium hydrogen phosphate 0.3 M, 1:1:1). The reagents are let react for 1 min before adding 5 µL of glacial acetic acid and shaking on vortex to stop it. The vial is let stand 10 min before injecting. The derivatized extracts, stable for a maximum of 8 h at room temperature, were analyzed by a UPLC Nexera X2 assembled with a degassing unit (DGU-20A5R), a quaternary pump (LC-30AD), a refrigerated autosampler (SIL-30AC), a column oven (CTO-20AC), and a fluorescence (RF-20AXS) detector (Shimadzu, Kyoto, Japan). The instrumental operating conditions are reported in Table 3 [25].

#### 3.2.2. Confirmatory Approach

The PSTs extraction and HPLC-FLD analysis were performed according to EN14526:2017 [28] as implemented by National Reference Laboratory for Marine Biotoxins (NRL-MB).

#### 3.2.3. Representative Toxins Selection

The derivatization reaction to light the fluorescence of the molecules performed with periodate is not selective, and it produces derivatized products of all the 14 toxins, yielding, for some of them (GTX1,4, NEO, dcNEO, dcSTX, dcGTX2,3, and GTX6), 2 or 3 peaks, therefore, among the oxidized products yielded for the same toxin, only one was chosen: the least interfered or the more sensitive.

Moreover, in the screening method, some analytes co-elute, and the 14 target molecules cannot be determined individually: seven groups of co-eluting toxins were identified, and, for each group, a “representative toxin” was chosen (Table 4). The selection criterion was “the worst case scenario”; therefore, for group 1 and 4, the molecule with the highest TEF and the least intense fluorescence signal was identified as representative (Figure S2). In case of group 2, the molecule with the highest TEF was chosen. The semiquantitative EURLMB SOP screening procedure identifies six groups of co-eluting toxins, and six molecules act as group “representative toxins”. The coelution is a declared limit of this approach; however, the overestimation of the screening method allows to control and compensate the eventual underestimation due to this problem. Moreover, in the here described approach, a seventh “representative toxin” was included: the dcNEO. In the EURLMB SOP, the dcNEO is quantified together with GTX6, NEO, dcSTX, taking the latter as “representative toxin”. Here was decided to extract the dcNEO from the original group and treat it independently because its primary oxidation product elutes in the chromatogram at a retention time only little interfered from the secondary oxidation product of the dcSTXs, and its signal is 12 times more intense than its secondary which is, although, considered in the of EURL SOP. This choice brings to a significantly clearer interpretation of the toxins profile and to a more sensitive method (Figure S1).

#### 3.2.4. PSTs Analytical Batch Building and Quantification

In Table 4, a full detailed description of the calculation carried out to implement the presence/absence screening method with cut-off is reported.

In the implemented presence/absence screening method, the 600 µg STX·2HCl eqv/kg of the semiquantitative EURLMB SOP was chosen as threshold. The 600 µg STX·2HCl eqv/kg is a concentration expressed in Toxic Equivalent Quantity (TEQ) which takes into account the Toxicity Equivalency Factor (TEF) of the single toxins in the calculation. The test enables the screening of the fourteen target PSTs (Table 4) included in the EURLMB SOP.

Namely, the total PSTs toxicity of 600 µg STX·2HCl eqv/kg was equally divided among the 14 toxins; thereafter, the contribution of each of the seven groups to the cut-off was calculated by summing up the single contribution of the co-eluting toxins of the group. From the group contribution to the cut-off (TEQ) was then calculated the corresponding toxin concentration in µg/kg. Finally, the spiking concentration (µg/kg) was modulated as a result of the toxin instrumental response, raising the concentrations of the molecules with lower sensitivity and decreasing the concentration of the more sensitive ones in order to keep the final balance to 600 µg STX·2HCl eqv/kg.

**Figure 2:**
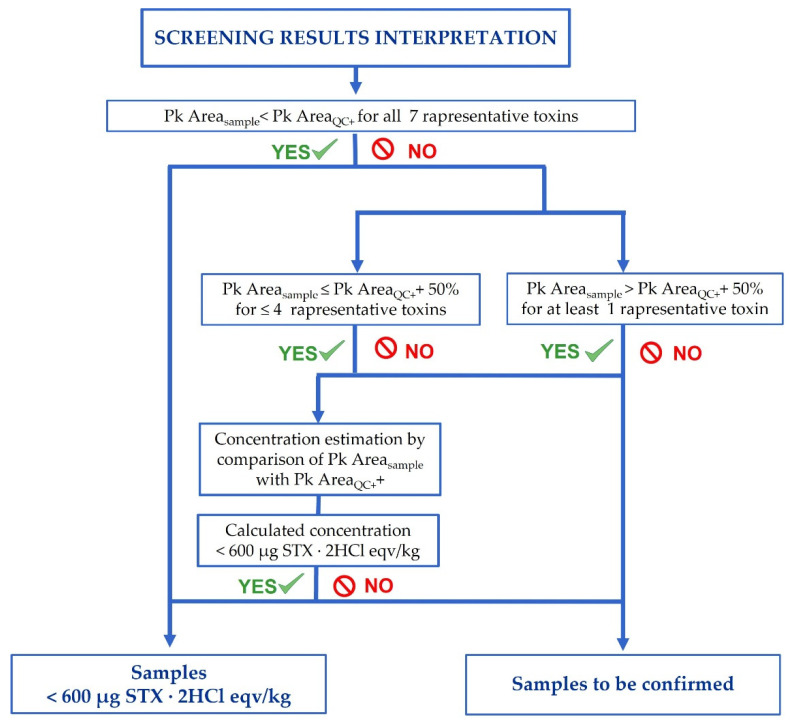
Flow diagram for PSTs screening results interpretation.

Each analytical batch includes the samples in analysis, a quality control positive sample spiked at the cut-off before the extraction (QC+), and a matrix-matched standard (MSd) prepared processing a blank sample throughout all the procedure and finally spiked with the standard mix of the seven “representative toxins” to obtain a concentration corresponding to the cut-off. An oyster tissue (*Crassostrea gigas*) was identified as blank matrix. The samples are analyzed by injecting both the oxidized and the non-oxidized extracts. The analysis of the non-oxidized extracts is required to identify possible naturally fluorescent molecules present in the matrix, which may interfere with the PSTs analysis and whose signals would need to be subtracted from the oxidized samples. In the results interpretation, no quantification is required. The chromatographic peak areas (Chr Pk area) are registered, and the samples identified as negative or suspect by comparison of the Chr Pk area measured in the sample for the single toxin and the Chr Pk area of the same toxin in the QC+. Criteria for results interpretation were defined and are described in detail in Figure 2.

In order to be able to quickly process the chromatographic raw data, an excel data sheet was developed. The data sheet is available for use within the present paper (File S3).

#### 3.2.5. Validation

The method validation was conducted on mussels at the STC (600 µg STX·2HCl eqv/kg) following the criteria for screening methods and performing replicated analysis (N = 5) on five different days [30], yielding a total of 25 independent experiments. In each analytical batch, together with the five spiked samples, also an MSd spiked at STC was prepared. The experiments enabled the calculation of the repeatability and reproducibility relative standard deviations (RSDr and RSDR) by ANOVA and recovery. The method was also validated in gastropods performing repeated analysis in repeatability condition (N = 5, 1 day). The results thus obtained were compared with those of mussels at the same concentration, obtaining satisfactory results.

In order to assess the fit for purpose of the procedure, we had to demonstrate that its precision is suitable for screening taking into account the chosen STC and considering that the PSTs maximum limit is 800 µg STX·2HCl eqv/kg.

Again, all the calculations were performed directly on peaks areas, and no quantification was necessary to estimate precision and trueness. The RSDr and RSDR were calculated on the seven representative toxins Chr Pk areas as well as the recovery, which was derived for each toxin from the ratio of the Chr Pk area in the spiked sample and the Chr Pk area of the MSd.

#### 3.2.6. Quality Control

Batch-to-batch method performances and results reliability are guaranteed by strict quality control (QC). One surely blank and one fortified sample (QC+) are processed in each analytical batch. Repeatability is measured in every single batch by multiple injections of the MSd. Furthermore, the recovery factor is calculated in each batch. Control charts are built to monitor the batch-to-batch results, X-charts (Shewhart chart) are compiled with spiked sample recoveries, and R-Charts are built to measure the repeatability of double-analyzed spiked bivalve samples. The here described PSTs analytical method was also tested by participating proficiency schemes (PTs) organized by Quasimeme (Wageningen University and Research, Wageningen, The Netherlands) in 2021 and NRL-MB (Cesenatico, Italy) in 2020 and 2021. Moreover, the method was also applied in the frame of an inter-calibration organized by the NRL-MB in March 2021 to test both qualitative and semiquantitative screening pre-column derivatization HPLC-FLD approaches [25]. The NRL-MB distributed ten samples with different PSTs contamination levels, and each Official Control Italian Laboratory taking part in the trial had to report results on each of the sample.

#### 3.2.7. Field Samples

The screening method described was applied to the monitoring of PST in mussel (*Mytilus Galloprovincialis*) collected along the Marche coast (twenty breeding sites and seven natural cliffs) in the frame of official monitoring plans. Overall, 569 samples were collected in one monitoring year (March 2021–February 2022) and submitted to PSTs analysis with the screening procedure implemented.

## 4. Conclusions

Official laboratories need fast and high throughput analytical methods to assure food safety. The EN 14526:2017 reference method in the EU is too complex and time-consuming to screen out negative samples from the positives in periods and geographic areas in which PST levels are negligible. In the present paper, a quicker and further simplified presence/absence test with a cut-off of 600 μg STX·2HCl eqv/kg was implemented, validated, and accredited following UNI CEI EN ISO/IEC 17,025 2018. It is an efficient, high throughput tool to screen out samples with very low PST levels from samples needing confirmation from laboratories in charge of monitoring biotoxins contamination in mollusks. This approach allows replacing the MBA as imposed by Regulation EU N° 1709/2021 with an official HPLC-FLD method, as required by European legislation.

In the presented method, a seventh toxin was introduced with respect to the EURLMB SOP (dcNEO), enabling a clearer interpretation of the chromatograms and enhancing the sensitivity. It was toughly described the validation approach in order to help official laboratories in implementing the screening procedure. The method was applied and also validated in gastropods, obtaining comparable results with respect to mussels and expanding their applicability since gastropods are often included in the monitoring plans

The proposed approach was applied to 569 samples which all showed PST concentrations significantly lower with respect to the maximum level of 800 μg STX·2HCl eqv/kg. To the best of our knowledge, this is the first report of one full year screening of PSTs in mussels by HPLC FLD in Italy and one of the few in Europe. The reported data contribute to a better understanding of the geographic distribution and of the seasonality of the PSTs contamination phenomenon in mollusks.

## Figures and Tables

**Table 1:** Validation study results on mussels and gastropods. Application of Single factor ANOVA (only mussel).

TOXIN			N Samples	RSD_r_	R	N Samples	RSD_r_^1^	RSD_R_	R	n	t (one tail)	t × RSD_r_%	ML	STC Worst Case
				%			%				*p* = 0.01		(µg STX·2HCl eqv/kg)	
			Gastropods			Mussels								
	Spiked Concentration (µg/kg)	N days	1			5								
**GTX 1,4**	200		5	17	62	25	5.1	25	75	20	2528	19,285	**800**	**646**
**C1,2**	140		5	20	72	25	6.4	33	80					
**dcNEO**	100		5	8.1	56	25	4.2	28	67					
**dcSTX**	120		5	12	106	25	8.0	50	79					
**GTX2,3**	100		5	8.3	53	25	11	23	69					
**GTX5**	160		5	20	88	25	11	35	71					
**STX**	160		5	18	60	25	7.9	32	72					
		**MEAN**	**5**			**25**	**7.6**	**32**	**73**					

RSDr% = repeatability relative standard deviation, R% = recovery, RSD_R_% = reproducibility relative standard deviation, n = number of replicate, t = t of student, STC = screening target concentration.

**Table 2:** PSTs screening method proficiency tests (PTs) results.

	***Samples***	***Lab Result Semiquantitative Screening***	***Lab result Quant*** ***itative*** ***Extimation***	***PT Assigned*** ***Value***	***Z’**-**Score***
			*μg STX·2HCl eqv/kg*		
**NRLMB** **December 2020**	**CRM_20_P_01**	PSTs to be confirmed		>800	
	**CRM_20_P_02**	<600		<800	
	**CRM_20_P_03**	<600		<800	
**QUASIMEME** **July 2021**	**QST 302 BT**	PSTs to be confirmed	2271	1780	2.1
	**QST 303 BT**	PSTs to be confirmed	3075	2222	2.9
	**QST 304 BT**	PSTs to be confirmed	4272	2771	3.9
**NRLMB** **March 2021**	**I**	PSTs to be confirmed	1803	1280	
	**II**	PSTs to be confirmed	1029	719	
	**III**	PSTs to be confirmed	952	608	
	**IV**	PSTs to be confirmed	1204	633	
	**V**	PSTs to be confirmed	810	692	
	**VI**	PSTs to be confirmed	608	577	
	**VII**	PSTs to be confirmed	695	865	
	**VIII**	PSTs to be confirmed	870	853	
	**IX**	PSTs to be confirmed	706	706	
	**X**	PSTs to be confirmed	244	342	
**NRLMB** **December 2021**	**CRM_21_P_01**	PSTs to be confirmed		>800	
	**CRM_21_P_02**	PSTs to be confirmed		<800	
	**CRM_21_P_03**	<600		<800	

**Table 3:** HPLC-FLD conditions for PSTs analysis.

**HPLC**	Column	Supelcosil LC-18, 15 cm × 4.6 mm (i.d.), 5 µm (SUPELCO)		
	Mobile phase A	0.1 M ammonium formate, pH 6.0		
	Mobile phase B	0.1 M ammonium formate, pH 6.0 with 5% (*v*/*v*) acetonitrile		
	Flow rate	1.0 mL/min		
	Injection volume	100 µL		
	Injector temperature	±6 °C		
	Column temperature	±35 °C		
	Gradient	Time (min)	A (%)	B (%)
		0	100	0
		5	95	5
		9	30	70
		11	100	0
		15	100	0
**FLD Detector**	Wavelength	Excitation	340 nm	
		Emission	395 nm	
	Run time	15 min		

**Table 4:** Calculation of single toxin contribution to the cut-off (600 µg STX·2HCl eqv/kg) for the presence/absence screening method.

Coelution Group		Toxin	TEF [2]	Group Representative Toxin	TEF (Representative Toxin)	Single toxin Contribution to the Cut-Off	Group Contribution to the Cut-Off (TEQ)	Group Contribution to the Cut-Off (µg/kg)	Modulated ^1^ Group Contribution to the Cut-Off (µg/kg)	Recalculated Group Contribution to the Cut-Off (TEQ)
**1**	1	**GTX1**	**1**	**GTX1**	**1.0**	43	172	172	**200**	200
	2	**GTX4**	**0.7**			43				
	3	dc GTX2	0.2			43				
	4	dc GTX3	0.4			43				
**2**	5	**GTX2**	**0.4**	**GTX3**	**0.6**	43	86	143	**100**	60
	6	**GTX3**	**0.6**			43				
**3**	7	**GTX5**	**0.1**	**GTX5**	**0.1**	43	43	430	**160**	16
**4**	8	GTX6	0.1	**dcSTX**	**1.0**	43	129	129	**120**	120
	9	NEO	1			43				
	10	**dcSTX**	**1**			43				
**5**	11	**dcNEO**	**0.4**	**dcNEO**	**0.4**	43	43	108	**100**	40
**6**	12	**STX**	**1**	**STX**	**1.0**	43	43	43	**160**	160
**7**	13	**C1**	**0.1**	**C1**	**0.1**	43	86	860	**140**	14
	14	**C2**	**0.1**			43				
**TEQ**						**602**	**602**			**610**

^1^ The concentration of the reference toxin of the group was redefined as a result of the instrumental response.

## Data Availability

Not applicable.

## Supplementary Materials

The following are available online at www.mdpi.com/article/10.3390/molecules27154702/s1, Table S1: Mussels and gastropods sample chromatographic peak areas measured during validation. Table S2: Spiked mussels and gastropods samples recoveries during validation. Figure S1: Group representative toxin selection in case of coelution (Group 1 and 4 listed in Table 4). Figure S2: Standard Mix (MSd mix 7) chromatogram compared with single toxins chromatograms. Identification of first oxidation (in green) and secondary oxidation (in red) product peaks. File S3: Excel data sheet for PSTs screening analysis results elaboration.

## Sample Availability

Samples of the compounds are not available from the authors.
