# Supplementary material for: A High Throughput Screening HPLC-FLD Method for Paralytic Shellfish Toxins (PSTs) Enabling Effective Official Control
Source: Molecules. 2022 Jul 22;27(15):4702. doi: 10.3390/molecules27154702 (PMC9331220; doi:10.3390/molecules27154702)
Supplement: Supplementary file 1 [file molecules-27-04702-s001.zip › molecules-1821923 Supplementary materials.pdf]

molecules

MDPI

Supplementary Materials

A High Throughput Screening HPLC-FLD Method for Paralytic Shellfish Toxins (PSTs) Enabling Effective Official Control

Melania Siracusa [1], Simone Bacchiocchi [1], Alessandra Dubbini [1,*], Debora Campacci [1], Tamara Tavoloni [1], Arianna Stramenga [1], Martina Ciriaci [1], Sonia Dall'Ara [2] and Arianna Piersanti [1,*]

[1] Istituto Zooprofilattico Sperimentale Umbria e Marche "Togo Rosati", Via Cupa di Posatora, 3, 60131 Ancona, Italy; m.siracusa@izsum.it (M.S.); s.bacchiocchi@izsum.it (S.B.); d.campacci@izsum.it (D.C.); t.tavoloni@izsum.it (T.T.); a.stramenga@izsum.it (A.S.); m.ciriaci@izsum.it (M.C.)

[2] Fondazione Centro Ricerche Marine, Viale A. Vespucci, 2, 47042 Cesenatico, FC, Italy; sonia.dallara@centroricerchemarine.it

* Correspondence: a.dubbini@izsum.it (A.D.); a.piersanti@izsum.it (A.P.); Tel.: +071-41760 (A.D. & A.P.)

Table S1. Mussels and gastropods sample chromatographic peak areas measured during validation.

Mussels samples chromatographic peak areas of 5 samples spiked at total PST 600 µg STX eqv/kg analysed in 5 different days								
	I	II	III	IV	V	mean	SD	CV %
				I day				
GT1,4	118916	119590	110446	118657	113496	116221	4041	3.5
C1,2	62413	63375	57481	67116	68967	63870	4464	7.0
dcNEO	173456	172691	161963	169356	175099	170513	5217	3.1
dcSTX	140278	128480	122513	139093	134602	132993	7468	5.6
GTX2,3	516459	513401	469931	498191	511220	501840	19150	3.8
GTX5	21559	22452	21177	24407	25983	23115	2032	8.8
STX	90067	87046	85996	94200	100415	91545	5894	6.4
				II day				
GT1,4	119256	121697	120233	114765	113144	117819	3678	3.1
C1,2	68640	63924	62120	61117	62294	63619	2982	4.7
dcNEO	178409	170593	160417	154040	161166	164925	9577	5.8
dcSTX	145650	141020	131370	130796	134588	136684	6452	4.7
GTX2,3	575106	507817	477713	470305	468962	499980	44844	9.0
GTX5	24027	22994	21420	21125	22920	22497	1205	5.4
STX	80736	83598	74584	74469	82020	79081	4280	5.4
				III day				
GT1,4	96531	101245	97105	99778	98680	98668	1929	2.0
C1,2	40728	43757	43147	44157	43573	43072	1360	3.2
dcNEO	160965	157960	153608	141911	146894	152268	7842	5.2
dcSTX	118203	114427	121088	114817	117725	117252	2727	2.3
GTX2,3	477576	479955	446070	468725	503084	475082	20593	4.3
GTX5	17113	14986	15759	14845	15481	15637	904	5.8
STX	74455	68772	71300	65882	69781	70038	3164	4.5
				IV day				
GT1,4	90291	90577	101363	91662	92630	93304	4599	4.9
C1,2	64154	62478	59647	54387	51580	58449	5336	9.1
dcNEO	125765	120607	124741	122102	123853	123413	2066	1.7
dcSTX	81228	73723	69515	73032	72805	74060	4324	5.8
GTX2,3	423906	401936	366129	335117	438737	393165	42424	10.8
GTX5	18289	19744	18146	20746	18600	19105	1111	5.8
STX	63675	62963	62224	64697	62878	63287	941	1.5
				V day				
GT1,4	93146	90296	111975	90921	90349	95337	9373	9.8

C1,2	55212	55468	59065	54869	61067	57136	2775	4.9
dcNEO	142563	142015	146455	145922	136710	142733	3900	2.7
dcSTX	133631	127941	164352	142641	115044	136722	18406	13.5
GTX2,3	458527	439589	534399	413238	299953	429141	85139	19.8
GTX5	20977	19718	28629	25955	18462	22748	4346	19.1
STX	82972	78276	96479	86840	66351	82183	11097	13.5
**Gastropods samples chromatographic peak areas of 5 samples spiked at total PST 600 µg STX eqv/kg analysed in 1 day**								
	**I**	**II**	**III**	**IV**	**V**	**mean**	**SD**	**CV %**
**I day**								
GT1,4	84742	107050	80542	114890	82138	93872	15923	17.0
C1,2	36640	49175	36904	52662	34397	41955	8331	19.9
dcNEO	142902	165839	141778	162332	141067	150783	12224	8.1
dcSTX	151275	176024	147231	175102	130456	156018	19478	12.5
GTX2,3	324009	349304	310351	368692	303477	331167	27333	8.3
GTX5	25041	30265	24184	34371	20649	26902	5409	20.1
STX	62146	84488	62271	85855	62385	71429	12555	17.6

Table S2. Spiked mussels and gastropods samples recoveries during validation.

**Spiked Mussels recoveries %**								
	**I**	**II**	**III**	**IV**	**V**	**mean**	**SD**	**CV %**
**I day**								
GT1,4	82	83	76	82	79	80	3	3.5
C1,2	78	79	72	84	86	79	6	7.0
dcNEO	76	76	71	74	77	75	2	3.1
dcSTX	90	83	79	90	87	86	5	5.6
GTX2,3	84	84	77	81	83	82	3	3.8
GTX5	66	69	65	75	80	71	6	8.8
STX	74	72	71	78	83	75	5	6.4
**II day**								
GT1,4	80	82	81	77	76	79	2	3.1
C1,2	87	81	79	77	79	80	4	4.7
dcNEO	75	72	68	65	68	70	4	5.8
dcSTX	92	89	83	83	85	86	4	4.7
GTX2,3	88	77	73	72	71	76	7	9.0
GTX5	79	75	70	69	75	74	4	5.4
STX	80	82	73	73	81	78	4	5.4
**III day**								
GT1,4	71	75	71	73	73	73	1	2.0
C1,2	80	86	85	87	86	85	3	3.2
dcNEO	59	58	57	52	54	56	3	5.2
dcSTX	73	71	75	71	73	72	2	2.3
GTX2,3	67	67	62	66	70	66	3	4.3
GTX5	81	71	75	70	73	74	4	5.8
STX	72	66	69	64	67	68	3	4.5
**IV day**								
GT1,4	69	69	78	70	71	71	4	4.9
C1,2	87	84	81	73	70	79	7	9.1
dcNEO	83	79	82	80	81	81	1	1.7
dcSTX	80	72	68	72	71	73	4	5.8
GTX2,3	65	62	57	52	68	61	7	10.8
GTX5	61	66	61	69	62	64	4	5.8
STX	66	66	65	67	65	66	1	1.5
**V day**								
GT1,4	69	67	83	67	67	71	7	9.8

C1,2	75	75	80	75	83	78	4	4.9
dcNEO	54	54	55	55	52	54	1	2.7
dcSTX	76	73	94	81	66	78	11	13.5
GTX2,3	65	63	76	59	43	61	12	19.8
GTX5	68	64	92	84	60	73	14	19.1
STX	73	69	85	76	58	72	10	13.5
**Spiked Gastropods recoveries %**								
	**I**	**II**	**III**	**IV**	**V**	**mean**	**SD**	**CV %**
**I day**								
GT1,4	56	71	53	76	54	62	11	17.0
C1,2	63	85	64	91	59	72	14	19.9
dcNEO	53	61	53	60	52	56	5	8.1
dcSTX	102	119	100	119	88	106	13	12.5
GTX2,3	52	56	49	59	48	53	4	8.3
GTX5	82	99	79	113	68	88	18	20.1
STX	53	72	53	73	53	60	11	17.6

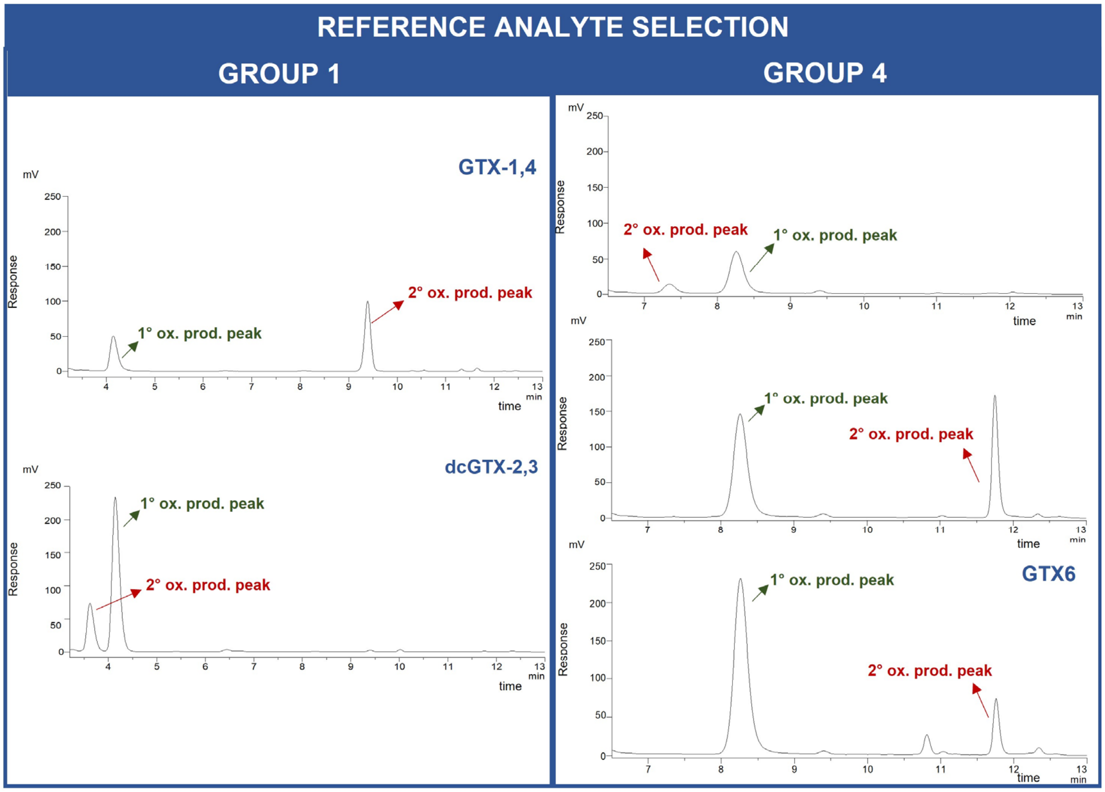

Figure S1. Group representative toxin selection in case of coelution (Group 1 and 4 listed in Table 4).

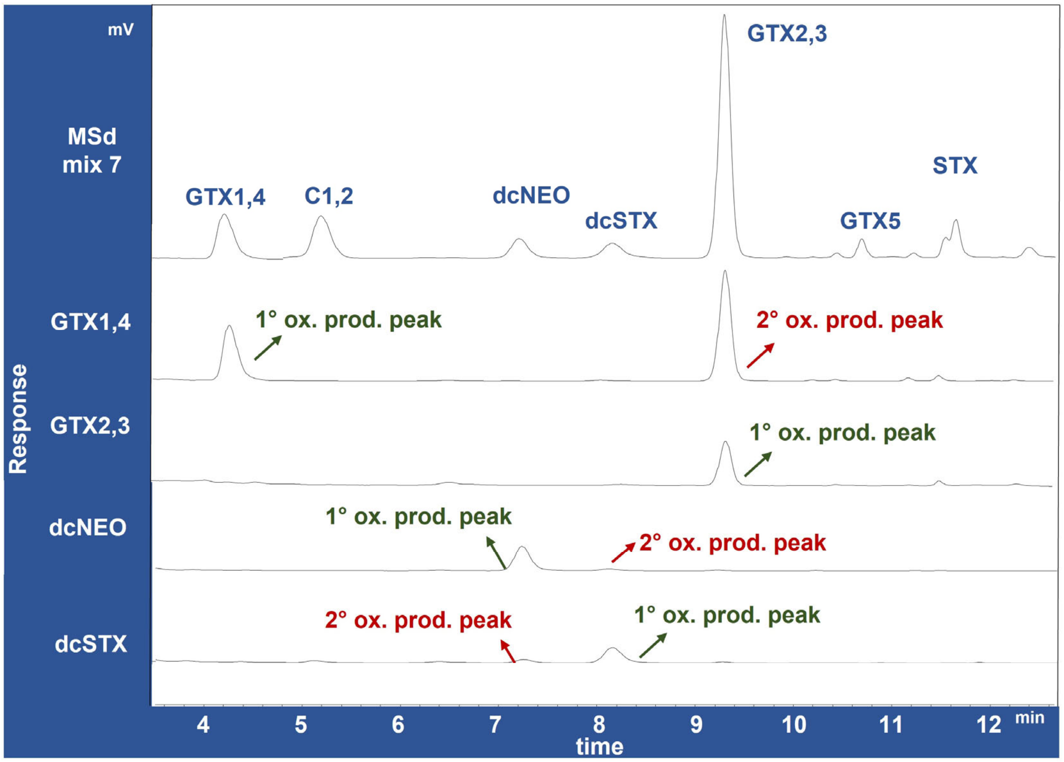

Figure S2. Standard Mix (MSd mix 7) chromatogram compared with single toxins chromatograms. Identification of first oxidation (in green) and secondary oxidation (in red) product peaks.
